# Sensorless vector-controlled induction motor drives: Boosting performance with Adaptive Neuro-Fuzzy Inference System integrated augmented Model Reference Adaptive System

**DOI:** 10.1016/j.mex.2024.102992

**Published:** 2024-10-05

**Authors:** Govindharaj I, Dinesh Kumar K, Balamurugan S, Yazhinian S, Anandh R, Rampriya R, Karthick G, Michael G

**Affiliations:** a Department of Computer Science and Engineering, Vel Tech Rangarajan Dr.Sagunthala R&D Institute of Science and Technology, Tamil Nadu 600062, India; b School of Computer Science and IT, JAIN (Deemed-to-be University), Karnataka 560069, India; c Department of Electronics and Communication Engineering, Vel Tech Rangarajan Dr.Sagunthala R&D Institute of Science and Technology, Tamil Nadu 600062, India; d Department of Artificial Intelligence and Data Science, Vel Tech Rangarajan Dr.Sagunthala R&D Institute of Science and Technology, Tamil Nadu 600062, India; e Department of Computer Science and Engineering (Artificial Intelligence), Madanapalle Institute of Technology & Science, Andhra Pradesh 517325, India; f Faculty of Engineering And Technology, Department of Computer Science and Engineering, Annamalai University, Tamil Nadu 608002, India; g Department of Computer Science and Engineering, Saveetha School of Engineering, Saveetha Institute of Medical and Technical Sciences, Saveetha University, Tamil Nadu 602105, India

**Keywords:** Adaptive Neuro-Fuzzy Inference System, Induction motor drive, Load variations, Model reference adaptive system, Rotor speed estimation, Sensorless induction motor, Speed control, Integration of Adaptive Neuro-Fuzzy Inference System (ANFIS) with Model Reference Adaptive System (MRAS)

## Abstract

The Model Reference Adaptive System (MRAS) is effective for speed control in sensorless Induction Motor (IM) drives, particularly at zero and very low speeds. This study enhances MRAS's resilience and dynamic performance by integrating an Adaptive Neuro-Fuzzy Inference System (ANFIS) controller into sensorless vector-controlled IM drives. The research addresses challenges related to parameter uncertainties, load variations, and external disturbances through the combination of MRAS and ANFIS. The ANFIS controller enhances dynamic performance by adjusting its parameters based on the error between estimated and measured rotor speeds, which improves reference speed tracking and ensures smoother drive operation. This integration of ANFIS with MRAS reduces the sensitivity of the sensorless control system to parameter variations, such as changes in motor parameters or load torque, thereby enhancing system stability. The primary goal is to ma-intain stability and mitigate the impact of parameter variations on the sensorless control system. The proposed MRAS-ANFIS scheme was evaluated using MATLAB and compared with existing systems. Results show that the ANFIS-enhanced MRAS delivers superior dynamic performance and robustness, proving to be an effective solution for applications demanding precise speed control and high reliability.

• **ANFIS integration for improved control:** integrating an Adaptive Neuro-Fuzzy Inference System (ANFIS) with MRAS enhances the dynamic performance and resilience of sensorless Induction Motor (IM) drives, particularly at zero and very low speeds.
• **Increased stability and robustness:** The ANFIS controller adapts to parameter uncertainties, load variations, and disturbances, improving speed tracking and reducing sensitivity to motor parameter changes, thus enhancing system stability.
• **Superior performance Validated:** MATLAB simulations show that the ANFIS-enhanced MRAS outperforms existing systems, offering superior dynamic performance and robustness, making it ideal for precise speed control applications*.*

## Graphical abstract

**Figure:**
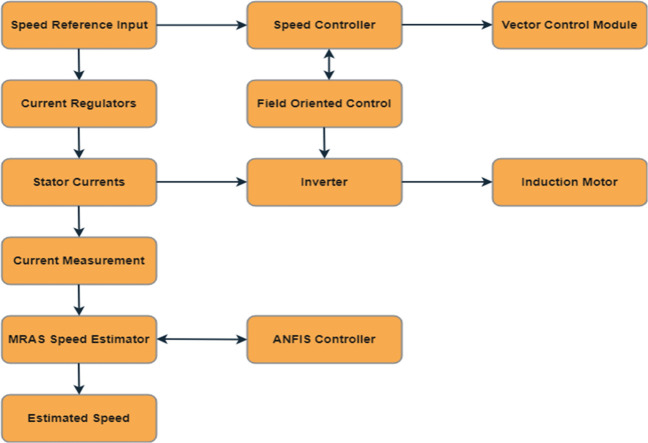

Specifications table

**Table:** 

Subject area:	Engineering
More specific subject area:	Control Systems Engineering - Sensorless Induction Motor Drives
Name of the reviewed methodology:	Integration of Adaptive Neuro-Fuzzy Inference System (ANFIS) with Model Reference Adaptive System (MRAS)
Keywords:	Adaptive Neuro-Fuzzy Inference System Controller; Induction Motor Drive; Model Reference Adaptive System Neural Networks; Sensorless Vector
Resource availability:	*N.A.*
Review question:	How does the integration of ANFIS with MRAS enhance the resilience and dynamic performance of sensorless vector-controlled Induction Motor drives, particularly in addressing parameter uncertainties, load variations, and disturbances?

## Background

### Introduction

One of the most widely utilized motors in applications these days is the AC induction motor. With the ability to transfer between 70 and 80 percent converts all electrical energy into mechanical form, induction machines represent the most significant class of industrial electrical machines. In the realm of electric cars, the training of Induction Motors with SMC has gained significant importance [1]. Sensorless control of Induction Motor (IM) drives has become a critical area of research due to the significant advantages it offers in terms of cost, reliability, and maintenance. To adopted approach for sensorless control, particularly effective at achieving accurate speed control at zero and very low speeds. However, traditional MRAS implementations often struggle with issues such as parameter uncertainties, load variations, and external disturbances, which can adversely affect their stability and dynamic performance. Numerous investigations have substantially demonstrated the efficiency of the SMC, which is resilient against disruptions and fluctuation in parameters [2]. In the event of extremely powerful oscillations, which result in the chattering phenomena, it is still limited [3]. A mechanical sensor must be installed in order to measure the speed of both the electrical vehicle and the induction machine [4]. Nevertheless, there is an additional expense associated with the addition of this sensor that may be comparable to the machine's cost. It follows that a good way to solve this issue would be through sensorless control. Low speed, however, presents a challenge for speed estimate [5]. Researchers and engineers have known since the early work on the order sensor-less with state observers that under some operating situations, especially at low speeds, the control's performance declines [6].

The ANFIS controller is designed to improve the dynamic performance of the sensorless vector-controlled IM drive by adapting its parameters based on the error between the estimated and measured rotor speeds. This adaptive mechanism ensures better tracking of the reference speed and smoother operation of the drive system. Furthermore, the proposed MRAS scheme with ANFIS reduces the sensitivity of the sensorless control system to parameter variations, such as changes in motor parameters or load torque.

## Literature survey

The improving the ANFIS's sliding mode performance might be accomplished with an ‘MRAS sensorless speed controller for induction motor drives linked to the IFOC technique’ [7]. The sliding mode and the ANFIS approach are combined to form the control strategy. The MRAS sensorless technique connected to the ANFIS system is used to assess the speed of the IM. High tracking efficiency, appropriate performance, robustness, and precision characterize this controller. Model Reference Adaptive Systems (MRAS) are widely utilized in sensorless vector control of induction motors (IM) due to their ability to estimate rotor speed accurately without the need for physical speed sensors. MRAS techniques have been extensively studied for their effectiveness in various operating conditions, particularly at zero and low speeds where traditional sensor-based methods may fail. Studies have shown that MRAS can achieve high precision in speed estimation by comparing the output of a reference model, which represents the ideal behavior of the motor, with an adaptive model that adjusts its parameters based on the error signal derived from the differences between these models.

An adaptive proportional-integral (PI) speed controller that drives an Induction Motor (IM) powered by a solar generator via vector control [8]. The suggested method addresses the issue of induction motor rotor resistance variation, which may have a detrimental impact on the speed control's efficiency. The demonstrated [9] how the MRAS allows sensorless induction motor (IM) drives to be effectively controlled at zero and extremely low speeds. With the proposed adaptive SMO, rotor speed the stator current, and rotor flux, are estimated. The purpose of an adaptive SMO is to improve the effectiveness, robustness, and speed tracking among the estimated and actual speeds during extremely low speed operation. Despite the advantages, MRAS implementations face significant challenges, especially regarding parameter uncertainties, load variations, and external disturbances. These factors can cause deviations in speed estimation and reduce the overall performance and stability of the control system. Several research works have focused on enhancing MRAS to mitigate these issues, such as introducing robust control strategies and adaptive algorithms that can cope with variations in motor parameters and load conditions.

A “novel general sliding mode controller for a DC-DC converter with output voltage regulation” [10]. Owing to the characteristics of some converters with non-minimum phases the output voltage is controlled indirectly. The output voltage error integral is used by a strong nonlinear controller to provide zero steady-state error. Adaptive Neuro-Fuzzy Inference Systems (ANFIS) combine the benefits of neural networks and fuzzy logic to form a powerful hybrid system capable of handling non-linearities and uncertainties in dynamic systems. ANFIS uses a fuzzy inference system (FIS) to model the control process with fuzzy rules and membership functions while employing neural networks to learn and adapt these rules based on input-output data. This adaptive capability makes ANFIS particularly suitable for control applications where the system parameters are not fixed and can vary over time.

To develop a novel technique based on the MRAS approach for predicting the sensorless indirect IFOC induction motor's rotor speed [11]. In actuality, this technique aims to lessen the sensitivity of the traditional MRAS to noise and system uncertainties while also improving its performance, particularly in low-speed areas. The goal of this endeavor is to achieve a high-performance vector-controlled induction machine drive by utilizing artificial intelligence (AI) to estimate the rotor speed through the use of an upgraded MRAS. An ANN structure serves as the foundation for the adjustable and reference models, which are intended to calculate approximately speed and rotor flux using recorded terminal voltages and currents. Integrating ANFIS with MRAS aims to leverage the adaptive learning capabilities of ANFIS to enhance the robustness and dynamic performance of MRAS-based sensorless vector-controlled IM drives. The literature indicates that hybrid approaches combining fuzzy logic with adaptive control systems can significantly improve performance in the presence of uncertainties and disturbances. ANFIS, by adapting its parameters based on the error between the estimated and actual rotor speeds, can provide more accurate speed estimation and better tracking of reference speeds, leading to smoother operation of the drive system.

A reliable non-linear controller for producing the zero steady-state errors and utilizes the integral of output voltage error [12]. Research has shown that incorporating ANFIS into MRAS can reduce the sensitivity of the control system to parameter variations such as changes in motor parameters or load torque. This integration results in a more stable and reliable control system that can maintain high performance even under varying *operating conditions. Studies* comparing ANFIS-enhanced MRAS with traditional MRAS approaches demonstrate significant improvements in speed tracking accuracy, robustness to parameter variations, and overall system stability. To build a grid-connected PV system with reference to photovoltaic cell modeling, DC-DC UK design, slider mode control, and other subjects [13]. Spectral analysis based and fundamental model-based techniques are the two basic types of speed estimation techniques [14]. Since, MRAS is one of the commonly used fundamental model depend speed estimation techniques in which motor speed and flux linkages is evaluated from stator voltages, currents. Difficulties like, flux pure integration, inverter nonlinearity, acquisition of stator voltage and current related to model-based IM drives operate at low speeds. This model-based method fails in low and the zero-speed operation for the rotor induced voltages are tiny or zero at stator frequency [15]. The literature has shown that the motor's dynamic performance is affected by changes in machine parameters due to temperature, stator, rotor resistances, magnetic saturation, and frequency [16]. Artificial Neural Networks (ANNs) are employed to solve most of the real-world problems [17]. Several hybrid AI like Neurogenetic, Neuro-Fuzzy genetic and Neuro –Fuzzy are used in power electronics and drives applications and space vector modulation using NN (Neural Networks) is also used for the control of IM drives [18].

Because of the self-learning capability, ANNs are used to represent a system without an accurate mathematical model. Artificial intelligence techniques like ANN and Fuzzy are used to induction motor fault diagnosis [19]. An ANN-based MRAS system is suggested the NN model replaced the adaptive model of the induction motor but pure integration problem and parameter variation problem are present and performance of the drive was affected. A two-layer NN to replace the induction motor's current model [20]. The NN with back propagation algorithm is utilized in predictive adaptive design and for flux estimate on IMD. Here, the NN is also used to replace VM to eliminate pure integration problem and the estimation of resistances in the rotor and stator.

MATLAB/Simulink is a common platform used for simulating and validating control systems, including MRAS and ANFIS. Numerous studies have employed MATLAB to model the dynamics of induction motors, implement control algorithms, and evaluate their performance under different scenarios. These simulations are crucial for comparing the proposed hybrid systems with existing methods and demonstrating their effectiveness before real-world implementation.

## Method details

**Fig. 1:**
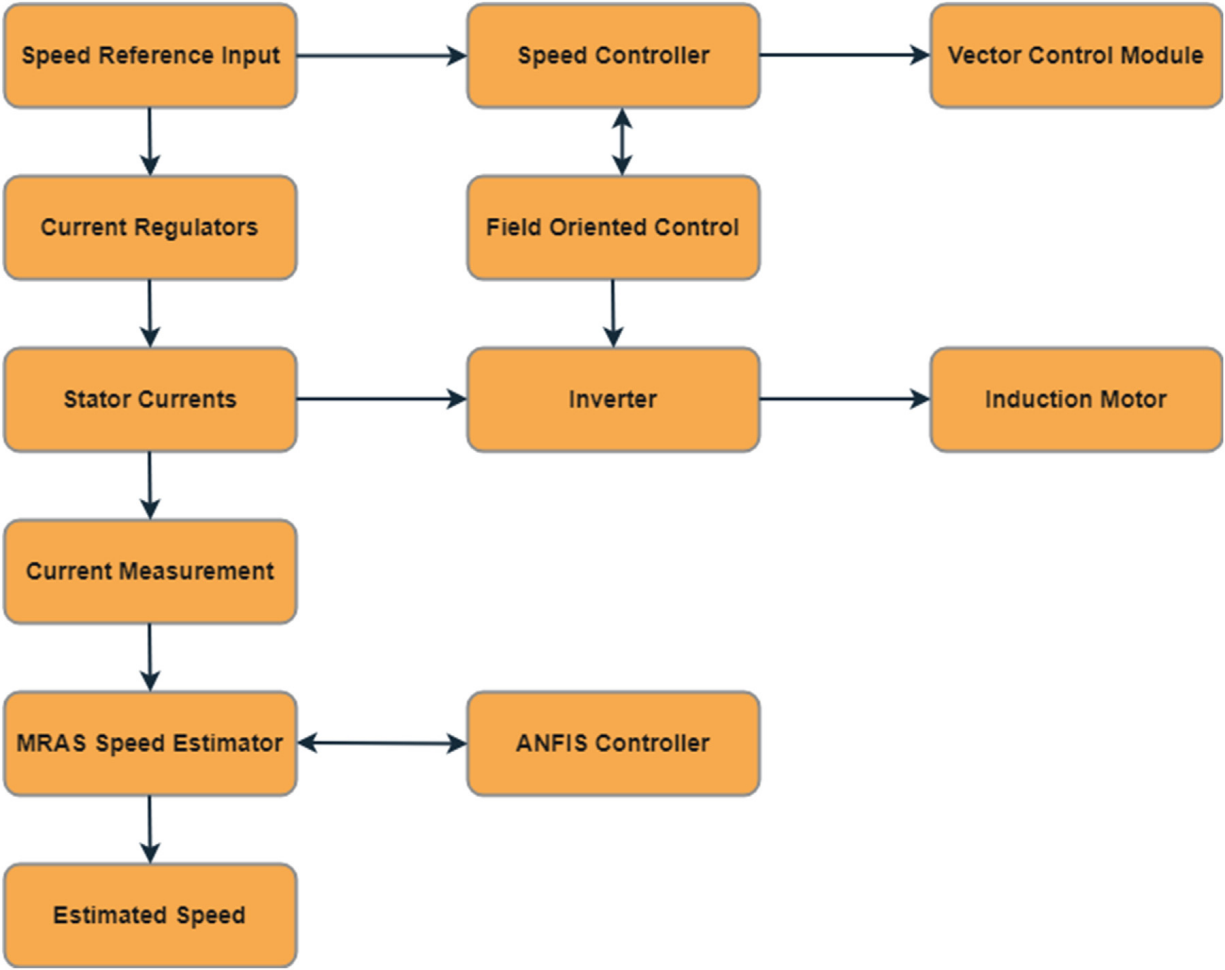
Proposed workflow model.

The usage of Neural Networks removes the requirement for a precise mathematical representation of a system, but selection of count of hidden layer neurons are a challenging task since it mostly affects the performance of system. Fuzzy logic controller is a non-linear optimizer, it can be used to a substitute for PI controller, but it requires human expertise and experience for the design of the controller. To overcome the drawbacks and limitations of fuzzy and NN controllers discussed so far, the ANFIS controller is proposed under this manuscript. The benefits of both fuzzy and ANN controllers are combined in the ANFIS controller, it acts as a robust controller. Induction Motor (IM) drives must be controlled in order to satisfy industry requirements for torque and speed. These devices do, however, have a commutator that corrodes, sparks, and needs regular repair. The advancement of digital signal processing and power semiconductor technology has made AC motor drives competitive substitutes for DC motor drives. However, because IM is a singly stimulated machine, controlling it is challenging. The Proposed workflow model shown in the below Fig. 1.

## System workflow

• Initialization: The system initializes the MRAS and ANFIS controller with initial parameters.
• Input Measurement: Stator currents and voltages are measured and fed into the vector control module.
• Vector Control Execution: The vector control module processes the inputs to generate the required current components for torque and flux control.
• MRAS Speed Estimation: The MRAS uses the stator current measurements to estimate the rotor speed, comparing the reference and adaptive model outputs to generate an error signal.
• ANFIS Adaptation: The ANFIS controller receives the error signal and adjusts the adaptive model parameters to minimize the error.
• Speed Control: The speed controller generates torque commands based on the difference between the reference speed and the estimated speed.
• Inverter Operation: The inverter converts these commands into appropriate voltages to drive the IM.
• Performance Monitoring: The system continuously monitors performance metrics and compares them to evaluate improvements over traditional systems.

**The contribution of this paper is described as follows:**

1. In an ANFIS controller is propose to exchange the conventional PI controller utilized on the Rotor Flux- Based Model Reference Adaptive System.
2. To demonstrate the effectiveness of the controller, the ANFIS based MRAS scheme is implement on the sensorless IM drive. The primary goal of the proposed controller is to develop the drive system robustness.
3. Accurate speed estimation was obtained by the proposed ANFIS based MRAS which helped to improve the dynamic characteristics of IMD when a reference values are altered suddenly.
4. The system simulation studies of 0.75 kW inductions motor carried out in MATLAB display that the proposed ANFIS based speed estimator performs well in enhancing the dynamic characteristics of the drive.
5. Moreover, comparisons of simulation outcome show the advantages of ANFIS controller such as small overshoot, high robustness, and minimum settling time.

## System design and integration

a. **Model Reference Adaptive System (MRAS) Setup:**

**Reference Model Development:** The reference model simulates the ideal dynamic behavior of the induction motor (IM) basedon predetermined motor parameters and stator current measurements.

**Adaptive Model Construction:** The adaptive model estimates the rotor speed by continuously adjusting its parameters to mini-mize the error with the reference model. The adaptation mechanism uses the error signal derived from the differences betweenthe reference and adaptive models.

b. **Adaptive Neuro-Fuzzy Inference System (ANFIS) Implementation:**

**Input Layer Design:** Inputs to the ANFIS include the error between estimated and measured rotor speeds, stator currents, andany other relevant operational variables.
**Fuzzy Inference System (FIS) Development:** The FIS is designed with fuzzy rules and membership functions that capture thenon-linearities and uncertainties of the control process.
**Neural Network Integration:** The neural network layer in ANFIS updates the parameters of the FIS based on learning algorithms,which adapt to changes in the input-output data.
**Output Layer Configuration:** The output layer generates control signals that adjust the adaptive model parameters in MRAS,aiming to minimize the error signal and enhance system performance.

## Vector control module

a. **Field-Oriented Control (FOC):**
  Decoupling Control Implementation: Field-oriented control is applied to decouple the torque and flux control in the IM, providing precise manipulation of motor dynamics.
  Current Regulators: Proportional-Integral (PI) controllers are used to regulate the stator current components (i_d and i_q), ensuring the desired levels of flux and torque.
b. **Speed Control Loop & Speed Reference Input:**
  Setpoint Specification: Define the desired speed setpoint for the IM drive system, which acts as the target for speed control.
c. **Speed Controller:**
  PI Controller Utilization: A PI controller generates torque commands based on the discrepancy between the reference speed and the estimated speed, aiming to reduce this error.

## Inverter and motor drive

a. **Pulse Width Modulation (PWM):**
  Signal Conversion Mechanism: PWM converts the control signals into appropriate voltage and frequency inputs that drive the IM, ensuring efficient motor control.
b. **Power Stage Configuration:**
  Electrical Power Supply: The inverter's power stage supplies the necessary electrical power to the IM based on the PWM signals, ensuring the motor operates as intended.

## Configuration of sensor less vector controlled induction motor drive

**Fig. 2:**
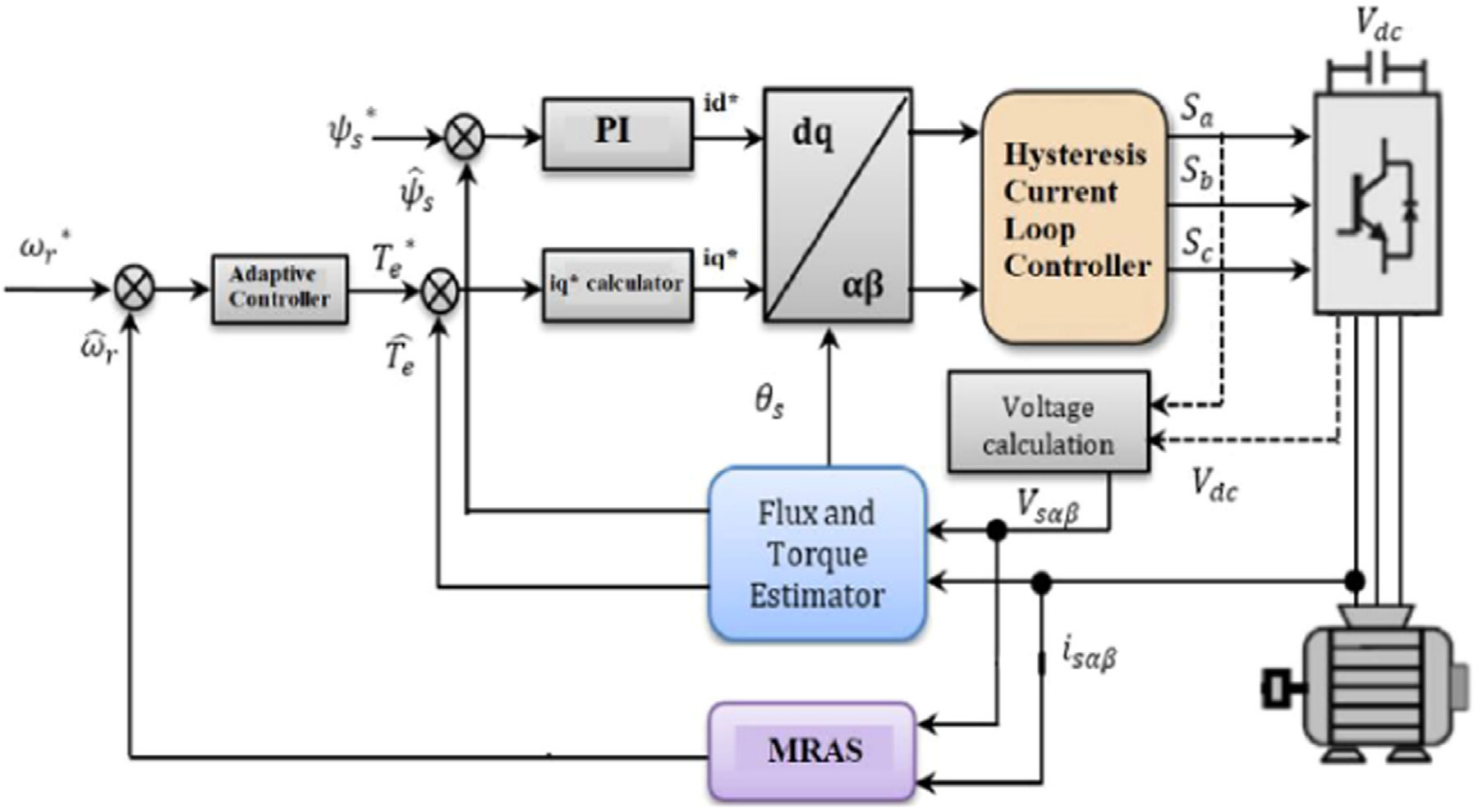
Configuration control of IM with MRAS module for speed estimation.

Fig. 2 shows the proposed MRAS neuro-fuzzy observer integrated into the overall structure of the system control block diagram for sensorless control IMs with ANFIS controller. Vsd.eq, Vsq.eq, and Vsd.n, Vsq.n are calculated by a SMC, and Vsd.n, Vsq.n by a Neuro-Fuzzy inference mechanism (ANFIS) [[3], [4]]. The control system scheme consists of three ANFIS controllers, PWM, MRAS observer, sliding-mode controller, and vector control transformation. Asymptotical stability is guaranteed by the control scheme principle, which can force the dynamics of the IMDs into a predefined sliding surface in a finite amount of time.

## Modeling of IM drive

The modeling of IM is necessary for torque and flux control [21]. By using the stationary reference frame, the motor is modeled. By using the dynamic modeling, the performance characteristics of motor is analyzed. The modeling of IM considered the variables like voltage, current, frequency, and speed. The stator and flux connection matrix are defined as follows: k num rotor bars and k num stator circuits in the common IM [22].

(1) $$\left[V_{Sta}\right]=\left[R_{Sta}\right]\left[I_{Sta}\right]+\frac{d\left[\zeta_{Sta}\right]}{dt}$$

(2) $$\left[V_{Rot}\right]=\left[R_{Rot}\right]\left[I_{Rot}\right]+\frac{d\left[\zeta_{Rot}\right]}{dt}$$

(3) $$\left[\zeta_{Sta}\right]=\left[L_{l,Sta}\right]\left[I_{Sta}\right]+\left[L_{l,Rot}\right]\left[I_{Rot}\right]$$

(4) $$\left[\zeta_{Rot}\right]=\left[L_{l,Sta}\right]\left[I_{Sta}\right]+\left[L_{l,Rot}\right]\left[I_{Rot}\right]$$

where, $V_{Sta}$ is denoted as the stator voltage, $R_{Sta}$ is denoted as the stator resistance, $I_{Sta}$ is denoted as the stator current, $\zeta_{Sta}$ is denoted as the stator flux, $V_{Rot}$ is denoted as the rotor voltage, $R_{Rot}$ is denoted as the rotor resistance, $I_{Rot}$ is denoted as the rotor current, $\zeta_{Rot}$ is denoted as rotor flux, $L_{l,Sta}$ is denoted as stator leakage resistance, $L_{l,Rot}$ is denoted as the rotor leakage resistance

(5) $${\left[I_{Sta}]=[I_{Sta1}I_{Sta2}\cdots I_{Sta,k}\right]}^{T}$$

(6) $${\left[I_{Rot}]=[I_{Rot1}I_{Rot2}\cdots I_{Rot}\right]}^{T}$$

(7) $${\left[V_{Sta}]=[V_{Sta1}V_{Sta2}\cdots V_{Sta,k}\right]}^{T}$$

If IM refers, squirrel-cage machine then $[V_{Rot}]=0,$ ${\left[L_{Rot,Sta}\right]=\left[L_{Sta,Rot}\right]}^{T}$. The mathematical modeling in α-β axis of IM is defined as follows,

(8) $$\frac{d\zeta_{\alpha Sta}}{dt}=-R_{Sta}I_{\alpha Sta}+v_{\alpha Sta}$$

(9) $$\frac{d\zeta_{\beta Sta}}{dt}=-R_{Sta}I_{\beta Sta}+v_{\beta Sta}$$

_Flux linkage of the stator is described by,_

(10) $$\zeta_{Sta}=\frac{L_{Mag}}{L_{Rot}}\zeta_{Rot}+\frac{I_{Sta}}{\gamma L_{Rot}}$$

(11) $$\gamma =\frac{1}{\left(L_{Sta}L_{Rot}-L_{Mag}^{2}\right)}$$

where, magnetizing inductance is denoted as $L_{Mag}$,

(12) $$\frac{d\zeta_{\alpha Rot}}{dt}+R_{Rot}I_{\alpha Rot}+\omega_{Rot}\zeta_{\beta Rot}=0$$

(13) $$\frac{d\zeta_{\beta Rot}}{dt}+R_{Rot}I_{\beta Rot}-\omega_{Rot}\zeta_{\alpha Rot}=0$$

where, $\omega_{Rot}$$\omega_{Rot}$ is denoted as the rotor speed

The electromagnetic torque is described as,

(14) $$T_{EM}={\left[I_{Sta}\right]}^{T}\frac{\partial \left[L_{Sta,Rot}\right]}{\partial \varphi }\left[I_{Rot}\right]$$

Depend on pole pairs,$\;T_{EM}$ described by,

(15) $$T_{EM}=\frac{3p_{p}}{2}\left(\zeta_{Sta}⊗I_{Sta}\right)$$

(16) $$\frac{d\omega_{Rot}}{dt}=-BJ^{-1}\omega_{Rot}+J^{-1}p_{p}\left(T_{EM}-T_{Load}\right)$$

(17) $$\frac{d\varphi }{dt}=\omega_{Rot}$$

where, $T_{EM}$refers electromagnetic torque, φ refers mechanical angle, $\omega_{Rot}$ is denoted as mechanic speed, $T_{Load}$ as load torque, coefficient of friction is denoted as B, pole pairs is denoted as $p_{p}$, stator α−β component current is denoted as $I_{\alpha Sta}$, $I_{\beta Sta}$,stator α − β component flux is denoted as $\zeta_{\alpha Sta}$, $\zeta_{\beta Sta}$, rotor α – β component current is denoted as $I_{\alpha Rot}$,$\;I_{\beta Rot}$, and rotor α − β component flux is denoted as $\zeta_{\alpha Rot}$, $\zeta_{\beta Rot}$.

## Model reference of an adaptive system modeling

Based on the rotor flux of the machine, the MRAS method is chosen since it offers superior performance over alternative approaches and is also easier to apply in real-world scenarios. For constructing the rotor flow, two models are chosen:

**Reference model:** This makes benefit of the machine's stator equations and is not directly dependent on speed.

(18) $$\left\{ \begin{array}{c} \frac{d}{dt}\varphi_{rd}=\frac{L_{r}}{L_{m}}v_{sd}-\sigma L_{s}\frac{d}{dt}i_{sd}-R_{s}i_{sd}\\ \frac{d}{dt}\varphi_{rq}=\frac{L_{r}}{L_{m}}v_{sq}-\sigma L_{s}\frac{d}{dt}i_{sq}-R_{s}i_{sq} \end{array}\right.$$

**Adaptive model:** This model employs the machine's rotor equations and is clearly dependent on speed.

(19) $$\left\{ \begin{array}{c} \frac{d}{dt}{\hat{\varphi }}_{rd}=\frac{L_{m}}{\tau_{r}}i_{sd}-\frac{1}{\tau_{r}}{\hat{\varphi }}_{rd}{}_{rd}+\left(\omega_{s}-p\Omega_{est}\right){\hat{\varphi }}_{rd}{}_{rq}\\ \frac{d}{dt}{\hat{\varphi }}_{rd}{}_{rq}=\frac{L_{m}}{\tau_{r}}i_{sq}-\left(\omega_{s}-p\Omega_{est}\right)\left({\hat{\varphi }}_{rd}{}_{rd}-\frac{1}{\tau_{r}}{\hat{\varphi }}_{rd}{}_{rq}\right) \end{array}\right.$$

The Popov hyper stability criterion establishes the estimator's stability. The speed expression estimate can be expressed as follows:

(20) $$\hat{\Omega }=K_{p}e+\int K_{i}edt$$

**Improve Dynamic Performance of ANFIS based MRAS System for Sensor-less Vector Controlled IM Drive**

In this section, proves the better dynamic performance of ANFIS based MRAS system for sensorless vectors-controlled induction motor drive.

## Neuro-Fuzzy control

The presentation of the Neuro-Fuzzy ANFIS combines Neural networks' capacity for learning with the knowledge representation of fuzzy logic to provide concepts for controlling and observing different aspects of the IM system parameters. This work presents the construction of the control strategy employing the concepts of the ANFIS control scheme for the regulation of the speed observer, Vsd.n, and Vsq.n, among other parameters of the induction machine. The error and the error change are inputs used by the ANFIS controller. The second controller ANFIS's Vsq.n, the third controller ANFIS's speed observer, and the first controller ANFIS's modified reference Vsd.n are the outputs. The neural network block is linked to the rule base block [23]. The NN is trained to choose the appropriate collection of rule bases using the back-propagation algorithm. The ANFIS controller receives the inputs that have been fuzzified using the fuzzy sets. The back-propagation algorithm has the benefit of being simple to comprehend and having a wide range of successful applications [24]. This method is based on error back propagation and computes the weight changes layer by layer, beginning with the previous layer and working backward [25]. For control, the error back-propagation training algorithm-based neuro-fuzzy system is employed [26]. The final approach uses a mean square error cost function reduction through the use of the gradient descent search strategy [[27], [28]].

The neural network's weighting vector is changed to complete the minimization procedure [29]. The function below illustrates the error among the desired output and the network output, which is the cost function that is being minimized:

(21) $$F=\frac{1}{2}\sum_{j}f_{j}^{2}\left(k\right)=\frac{1}{2}\sum_{j}{\left[a_{j}^{*}-a_{j}\left(k\right)\right]}^{2}$$

where, $a_{j}\left(k\right)$ is denoted as the output of neuron j and $a_{j}^{*}\left(k\right)$ is denoted as the desired pattern for that neuron. At iteration number k, let $\eta_{ij}\left(k\right)$represent the learning rate parameter assigned to synaptic weight $w_{ij}\left(k\right)$.

The below equation lead to a sequence of updates the weight vector. The function listed below can be used to update the weights of the connections among 2 adjacent layers:

(22) $$\omega_{ji}\left(k+1\right)=\omega_{ji}\left(k\right)-\eta_{ji}\left(k+1\right)\frac{\partial F\left(k,w\right)}{\partial \omega_{ji}\left(k\right)}+\alpha \Delta \omega_{ji}\left(k\right)$$

where α, the momentum gain, is susceptible to local minima and needs additional processing in order to assess the gradient and k is the number of iterations, and $w_{ij}\left(k\right)$ is the weight change depending on the cost function's gradient F(k,w). The ANFIS controller receives the inputs that have been fuzzified using the fuzzy sets. Five network layers are used to construct the five functional blocks that make up the ANFIS: the database, rule base, defuzzification interface, decision making unit, and fuzzification interface.

• Layer 1: In this layer, each input node is associated with a particular input variable. Only input signals are sent to the second layer via these nodes.
• Layer 2: The membership function, also known as the fuzzification process, is carried out by each node.
• Layer 3: The rule layer is the name given to this layer. In other words, each node in this layer matches the preconditions of the fuzzy rules, determining out each rule's activation level. The count of layers in this layer is equal to the count of fuzzy rules. In these layers, every node calculates the normalized weights.
• Layer 4: As a de-fuzzifier, this layer is involved. All incoming signals are added together by the single node, represented by Σ.
• Layer 5: This layer, known as the output layer, converts the fuzzy classification outcome into a crisp by adding up all the inputs from layer 4.

There are two passes through the various network layers that make up the error back-propagation process: a backward pass and a forward pass. Assuming the precursor parameters remain stable, the first phase (forward pass) involves propagating the inputs layer by layer across the network and estimating the subsequent parameters using the least squares approach. The input samples are replicated in the second stage (backward pass), where the gradient descent method is used to modify the precursor parameters while presuming constant secondary parameters.

## MRAS with neuro-fuzzy controller

**Fig. 3:**
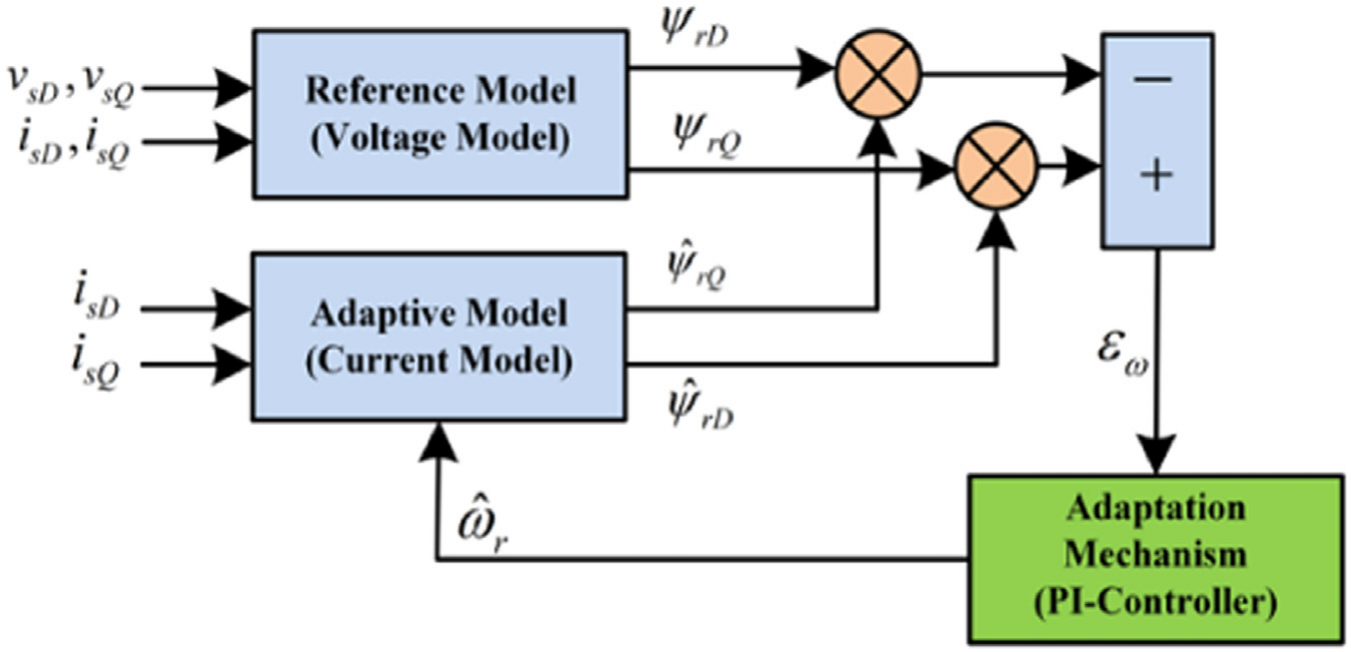
Structure of ANFIS - MRAS system.

The neuro-fuzzy MRAS observer depicted in Fig. 2 is shown in detail in the functional diagram shown in Fig. 3, above. Note that the flux error can only indicate when the control is in a steady-state situation. An offline learning approach is used to train the neuro-fuzzy network. The derivative of the flux error and flux error are the two inputs used by the NFC. Rotor speed is the result. The ANFIS controller and the NFC of the rotor speed are similar.

## Result and discussion

**Fig. 4:**
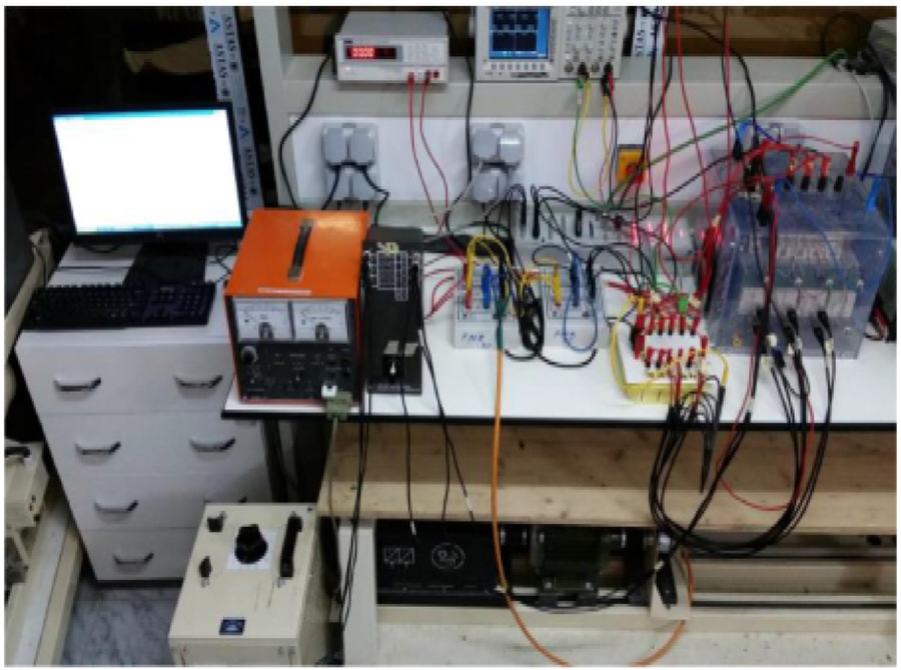
Experimental setup.

Fig. 4 displays the real-time experimental setup. From which, the linearized IMD with ANFIS and proportional integral torque controller can be examined. Real-time validation is performed on the proposed ANFIS-based linearized controlled IMD system. The real motor line voltages and currents are measured using Hall-effect current and voltage sensors. An A/D channel transmits the data to the DSP board. Speed encoders measure rotor speed. The DSP board yields the hysteresis current controlled the signals of pulse width modulation, which must be fed to the switches of the 3-phase voltage source inverter. An induction motor is attached to a direct current -motor shaft for obtaining the load perturbation necessary for analysing the torque. Next, the armature-circuit's load-shaft is altered by increasing resistance. An oscilloscope is used to observe all test variables, except for current.

**Fig. 5:**
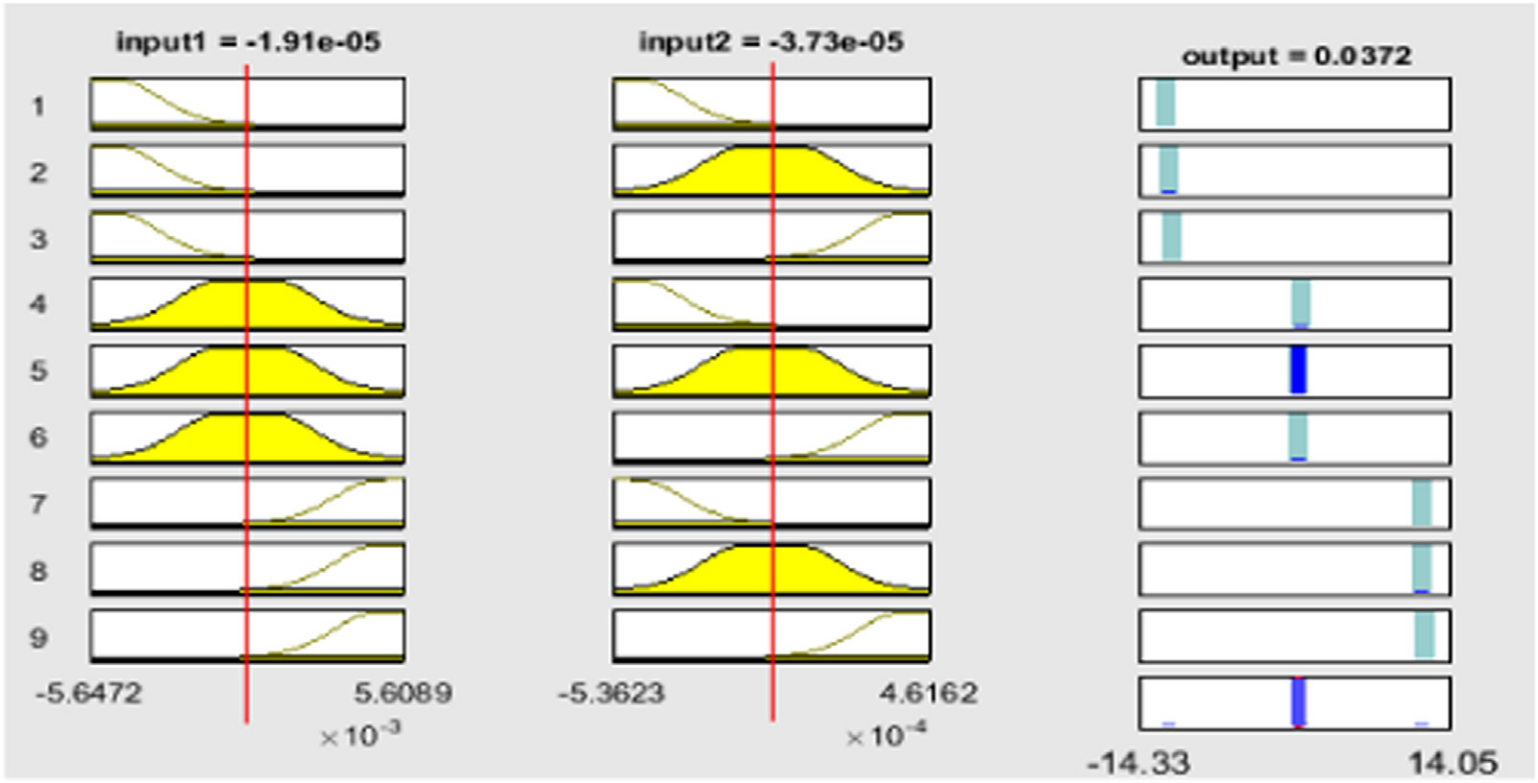
Rule viewer.

**Fig. 6:**
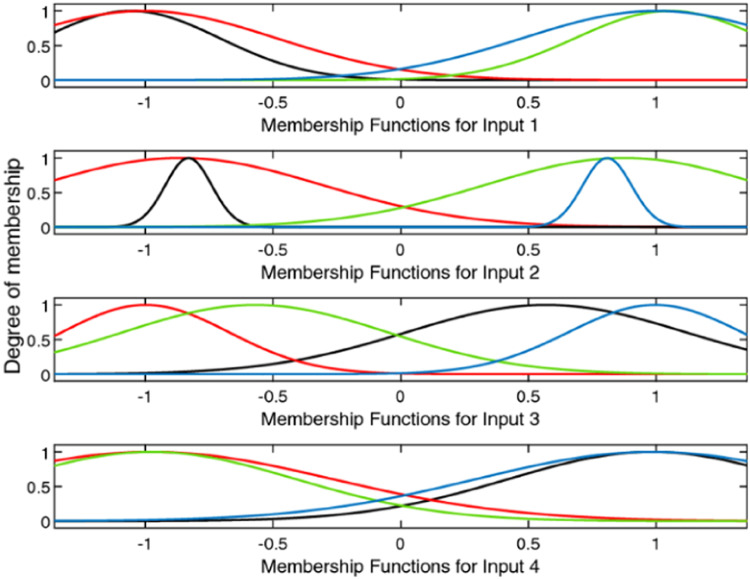
Membership functions of ANFIS system inputs designed to estimate extra torque.

**Fig. 7:**
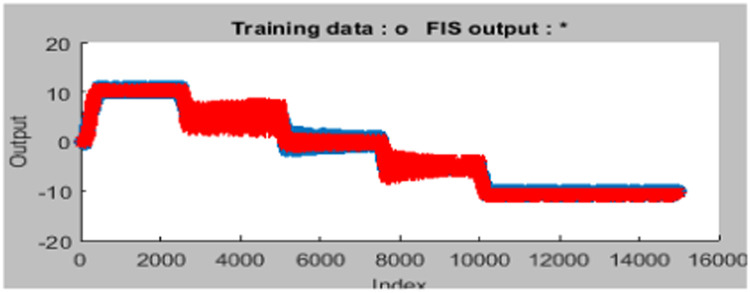
Training data in the ANFIS tool.

**Fig. 8:**
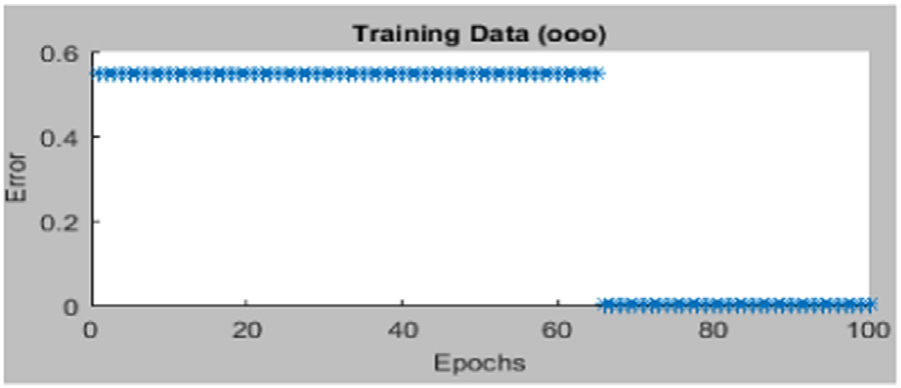
New trained data using ANFIS tuning tool.

**Fig. 9:**
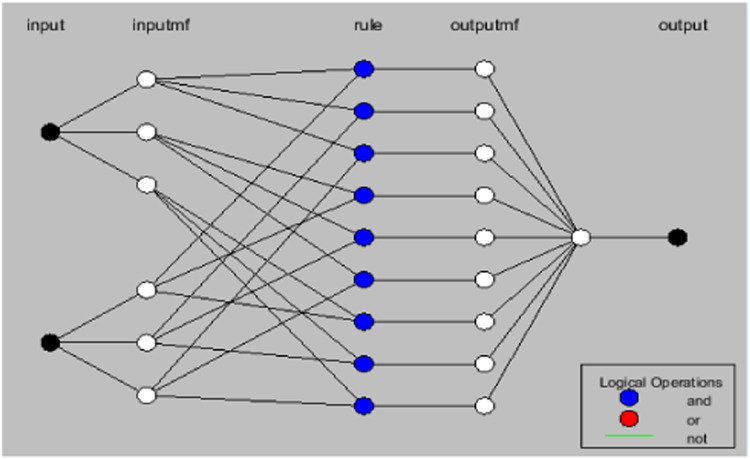
ANFIS model rule base structure.

**Fig. 10:**
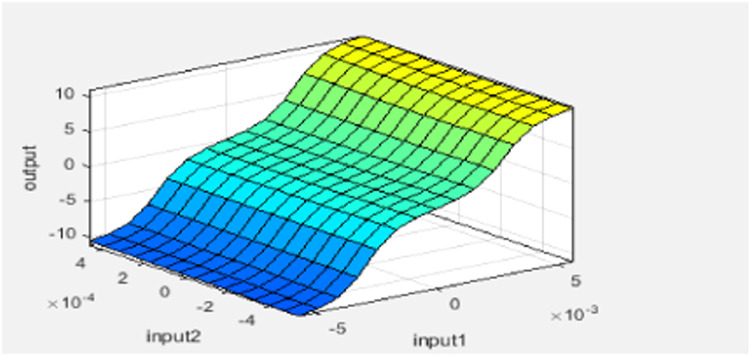
ANFIS surface viewer.

The design of the ANFIS controller does not require any human intervention for the development of fuzzy rules, i.e. rules are automatically developed from the given dataset is shown in Fig. 5. Gbell membership function is used for 2 inputs namely error, difference of the error is exposed in Fig. 6. Here, the ANFIS controller Training stage is displayed in Figs. 7 and 8 shows the completion of training and its output. Finally, the developed ANFIS structure and surface viewer are depicted in Figs. 9 and 10 respectively.

Here, the ANFIS based MRAS scheme is applied at sensorless vector-controlled IM drive and efficiency is estimated. Furthermore, the dynamic characteristics, robustness properties are presented, and then it is associated by using PI controller. The PI controller design coefficients are selected by trial, error method in like as, the output settled as is rapidly probable. The values are K_p_= 0.9, K_i_=8.1. Table 1 illustrates the ratings and parameters of the IM.

**Table 1:** Ratings and parameters of the IM.

Parameter	Values
Nominal Power P	0.75 kW
Frequency f	50 Hz
Voltage V	416 V
Rotor Resistance R_r_	7.5297 Ohm
Stator Resistance R_s_	12.6 Ohm
Stator leakage Inductance L_ls_	0.01671 H
Rotor leakage Inductance L_lr_	0.01671 H
Pole Pairs P	2
Mutual Inductance L_m_	0.53713 H
Moment of Inertia J	0.01225 kg m^2^
Rated Torque T_e_	5.1 Nm
Rated Speed N	1415 rpm
Rated Current I	1.8 A

The proposed ANFIS controller has two main objectives namely better dynamic performance like minimum settling time, peak overshoot, and robustness. Then, the proposed ANFIS based sensorless vector controlled IMD simulated using MATLAB/SIMULINK. Here, the speed overshoot and the settling time minimized below diverse operating regions including low and zero speed regions. To study this performance of ANFIS controller, four important tests performed on test drive, likened with a conventional PI controller. Here details and explanations of the tests presented.

**Test 1: Speed Command in without load at 50rpm**

Here, the test performed to evaluate a steady state efficiency of drive. To concentrate on performance of drive under less speed-operating region, a constant 50 rpm speed command is maintained throughout the test.

**Fig. 11:**
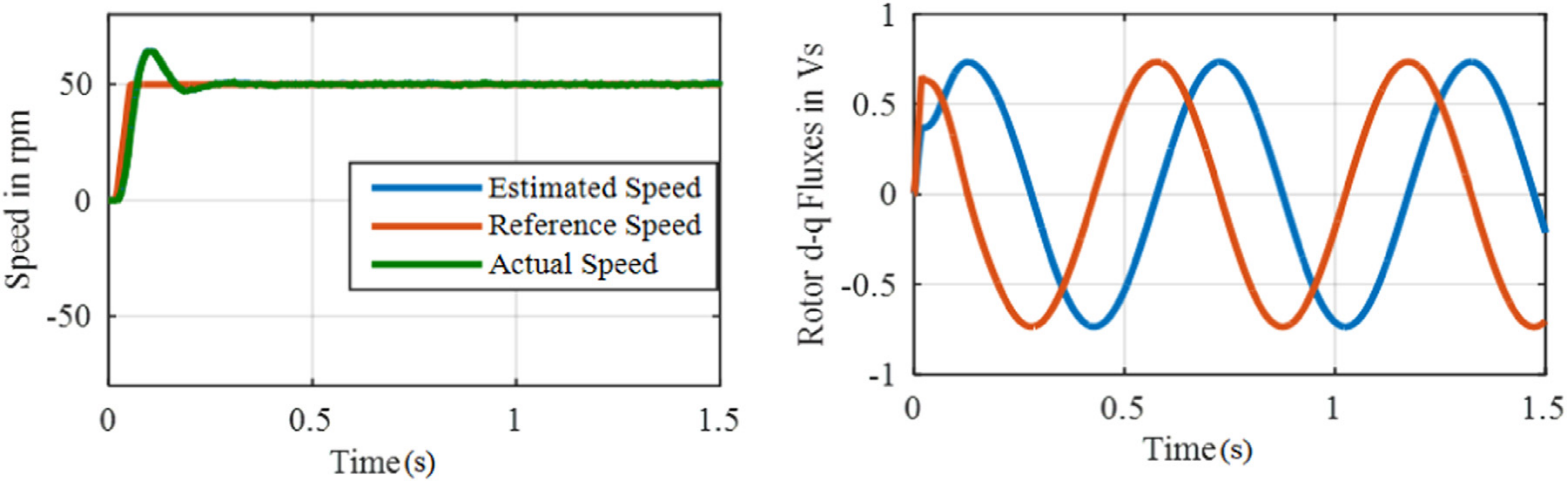
Performance under test1 with PI based MRAS.

**Fig. 12:**
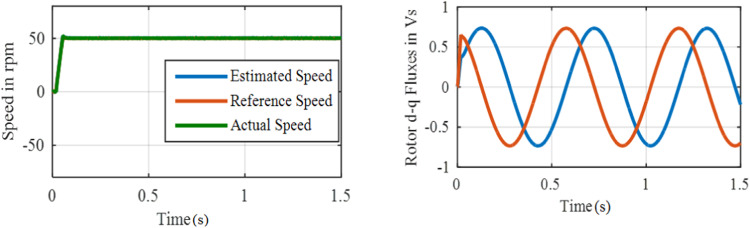
Performance under test-1 with ANFIS based MRAS.

Figs. 11 and 12 show the speed and flux waveforms for PI based and ANFIS based system correspondingly. For this PI based system, a 15-rpm overshoot is observed in a speed waveform, settling time is 0.28 s whereas a 2.5 rpm overshoot and 0.05 s settling time are observed with ANFIS based system. The results reveal that a stable operation with a negligible speed tracking error is possible with the ANFIS controller.

**Test 2: Speed Command 50rpm through 50** % **Load**

**Fig. 13:**
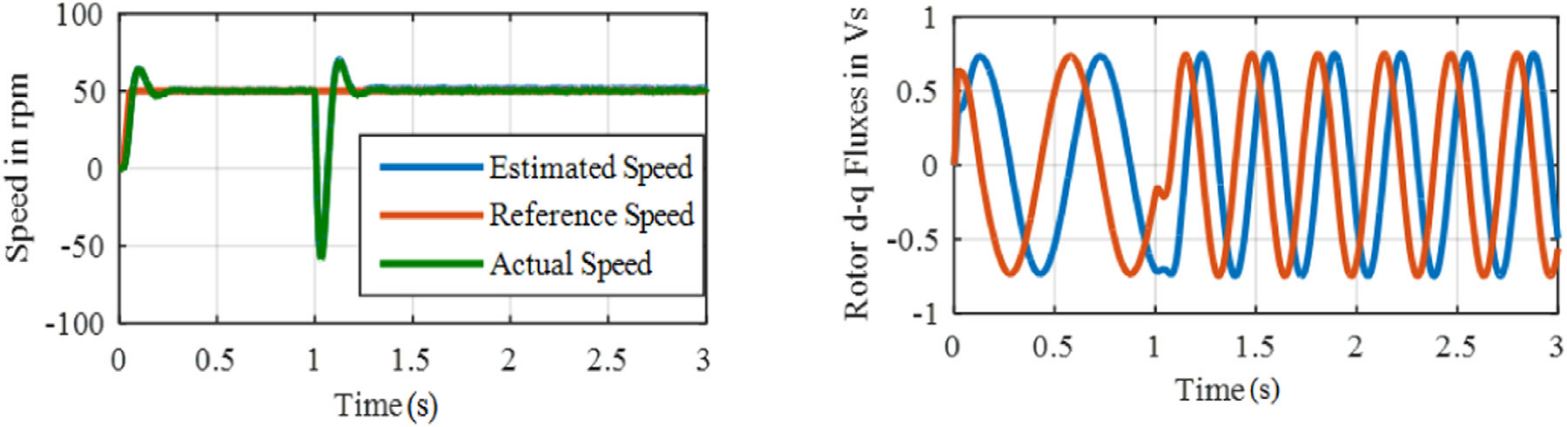
Performance under test2 with PI based MRAS.

The system performance of Steady-state is examined during the load condition. The drive is started with constant 50 rpm and at *t* = 1 s, 50 % of the rated torque is used to the motor. Performance under test 2 with PI based MRAS is shown in Fig. 13.

**Fig. 14:**
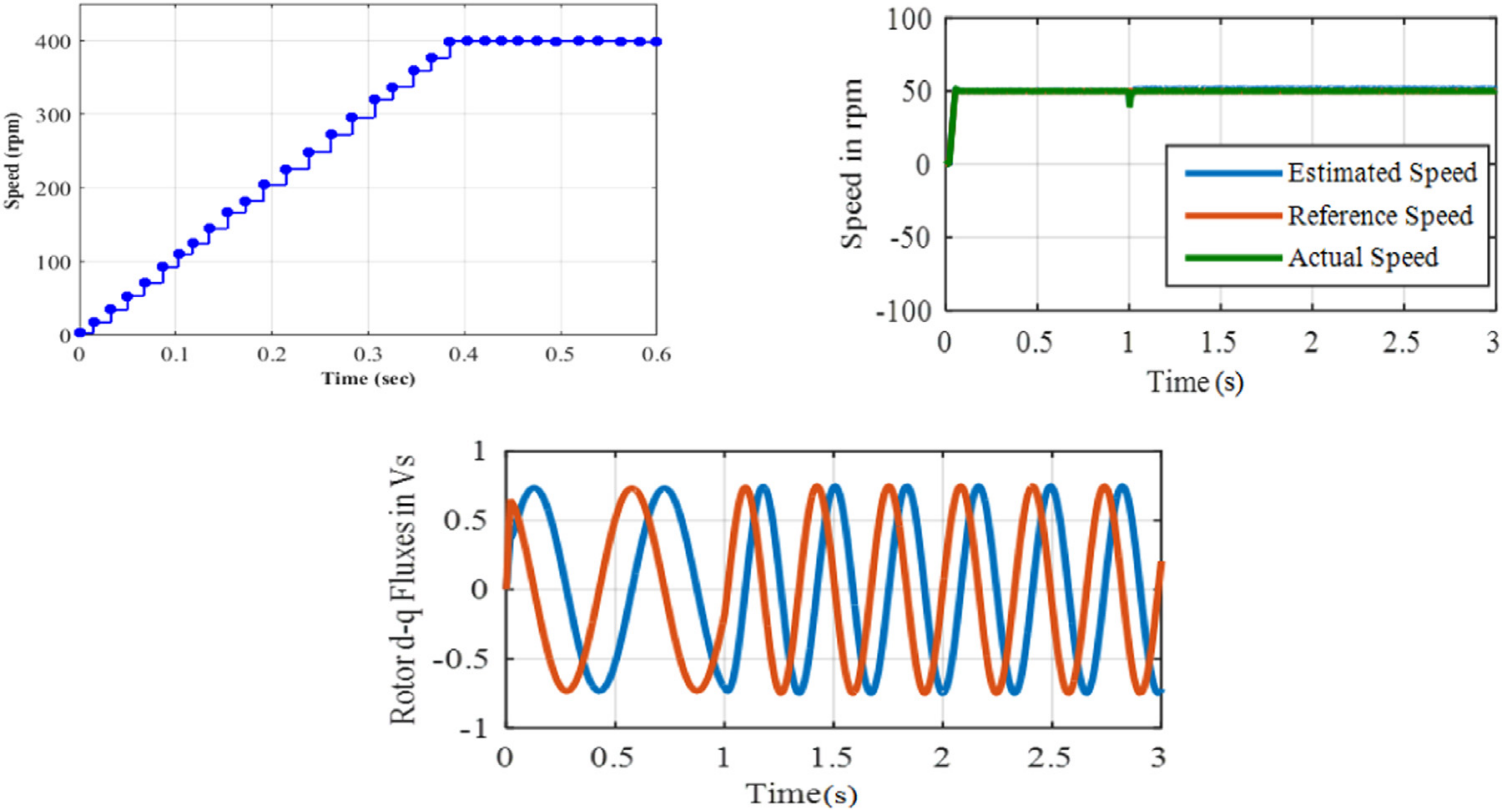
Performance under test2 with ANFIS based MRAS.

Performance under test 2 with ANFIS based MRAS is shown in Fig. 14. A Large undershoot of about 100 rpm and an overshoot of 20 rpm in speed are observed in PI based MRAS. The speed undershoot is large in the PI based system, whereas 10 rpm undershoot is observed with ANFIS controller. Hence, it's revealed that the proposed ANFIS based MRAS scheme can be used to develop the efficiency of the drive to track a reference value when a sudden change in load is used. At *t* = 1 s, once the load is engaging, the flux waveform is disturbed for the proportional integral controller and it is smooth in nature for ANFIS controller.

**Test 3: ± 50rpm including Zero Speed Operation**

**Fig. 15:**
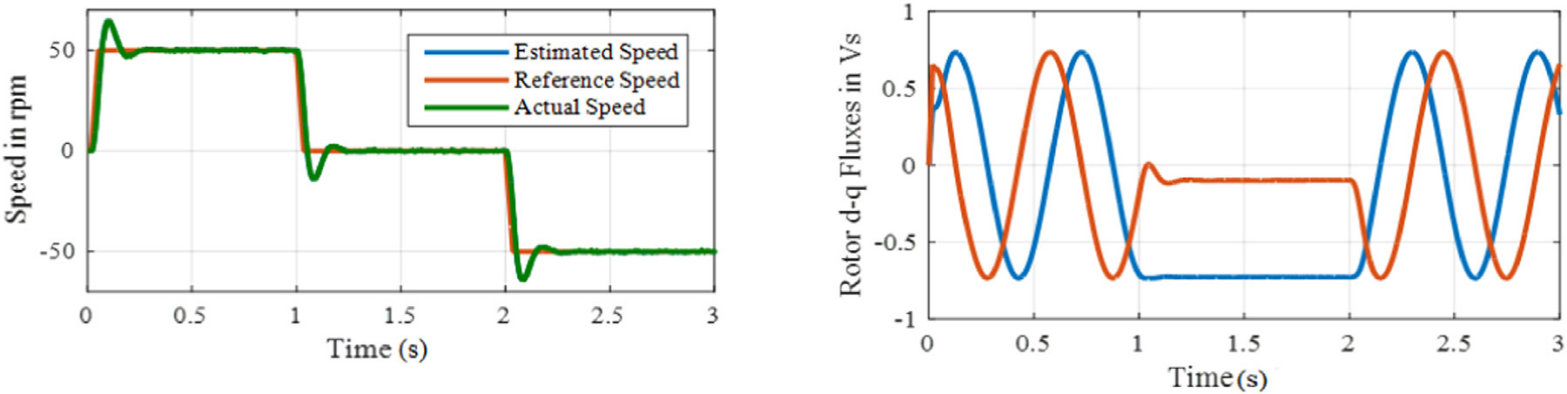
Performance under test3 with PI based MRAS.

**Fig. 16:**
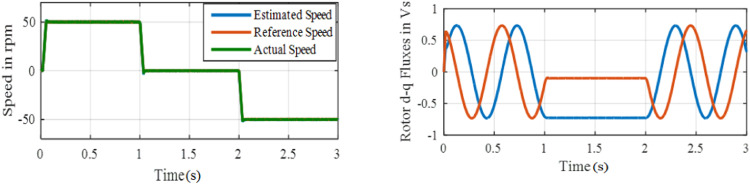
Performance under test3 with ANFIS based MRAS.

**Fig. 17:**
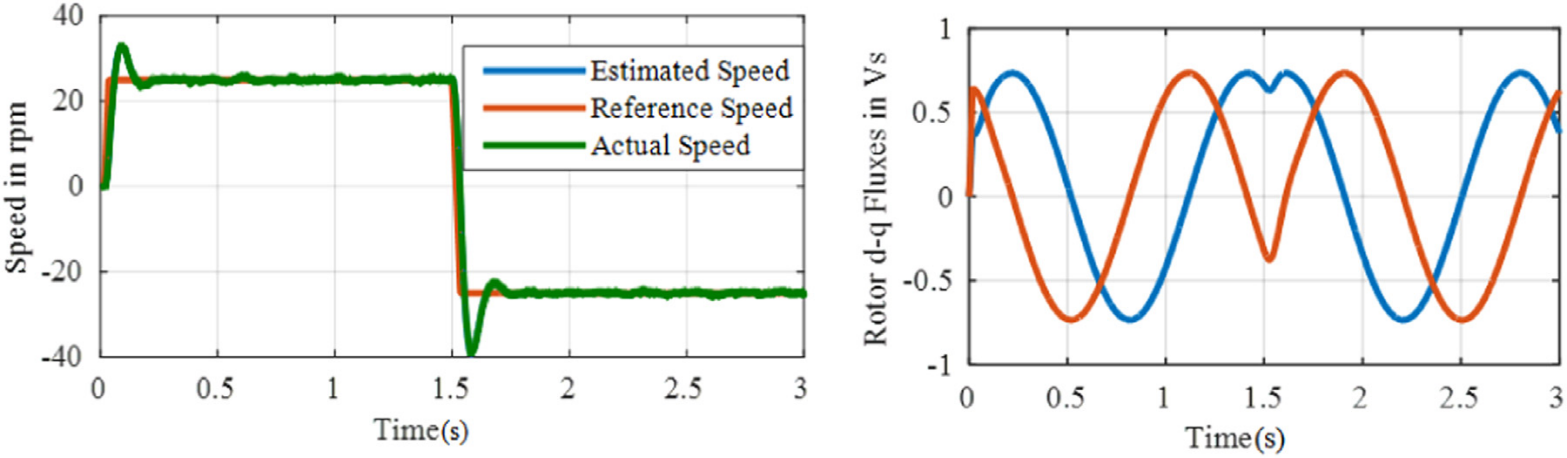
Performance under test 4 with PI based MRAS.

This test is performed to evaluate the robustness of drive to step changes on speed. To conduct the test, the speed command is changes from 50 to 0 rpm at *t* = 1 s and then it is changes to −50 rpm at *t* = 2 s. Figs. 15 and 16 shows speed and fluxes for proportional integral and ANFIS controller. The proposed controller is described in Fig. 17 that contains best performance, but it eliminated an overshoot during transients when reference values vary. Here, the speed overshoot is almost eliminated in ANFIS controller, whereas when PI controller is used, speed overshoot exists considerably and proper measures are to be taken to reduce that. Also, between *t* = 1 s and *t* = 2 s, there is a change in the flux waveform with PI controller, whereas for ANFIS controller, flux waveforms are smooth, as shown in Fig. 16.

Fig. 16 displays the Performance under test 3 with PI based MRAS. Fig. 17 displays the Performance under test3 with ANFIS based MRAS. Accurate speed tracking with negligible over and undershoot in speed are observed with the proposed ANFIS controller. The settling-time for proportional integral is 0.25 s, whereas 0.025 s is the settling time observed for ANFIS based system.

**Test 4: Speed Reversal Test with ±25rpm**

Here, the speed reversal capability of PI and ANFIS controllers compared to speed changes +25 to −25 rpm. Initially, the drive allowed to run at +25 rpm, a speed command is altered to – 25 rpm at *t* = 1.5 s.

**Fig. 18:**
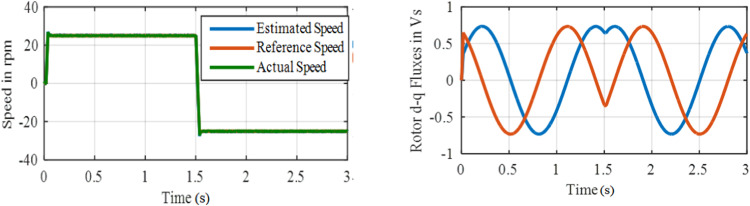
Performance under test4 with ANFIS based MRAS.

Performance under test 4 with PI based MRAS is displayed in Fig. 17. Performance under test4 with ANFIS based MRAS is displays in Fig. 18. The performance of this PI controller consists of large speed overshoot, whereas the proposed controller has negligible speed overshoot. The results show better speed reversal capability of the drive system with the ANFIS controller. An overshoot of 13 rpm and 0.22 s settling time are obtained in PI based system, whereas the overshoot for ANFIS based system is 3 rpm only.

**Test 5: Starting dynamics and forward motoring at 400rpm**

Here, while applying a DC-link voltage of 646 V, the motor accelerates steadily and attains its 400-rpm set point speed. The induction motor's speed, torque, current is set at 0.4 s, and the performance is determined by how each of these variables responds to changes in torque, speed, stator current and rotor flux.

**Fig. 19:**
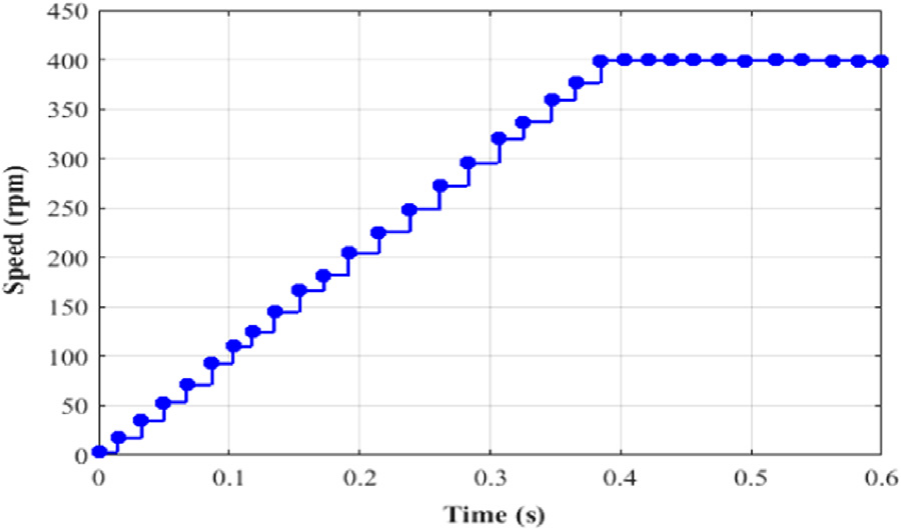
Performance of speed under test 5 with proportional integral based MRAS.

**Fig. 20:**
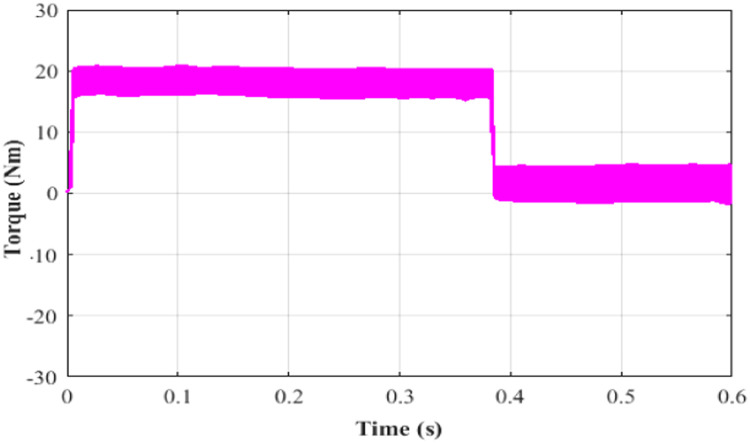
Performance of torque under test 5 with PI based MRAS.

**Fig. 21:**
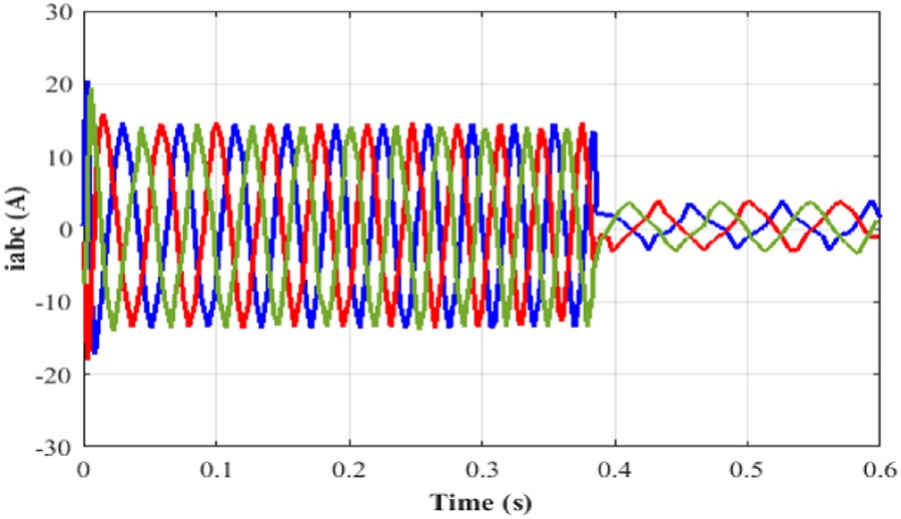
Performance of current under test5 with PI based MRAS.

**Fig. 22:**
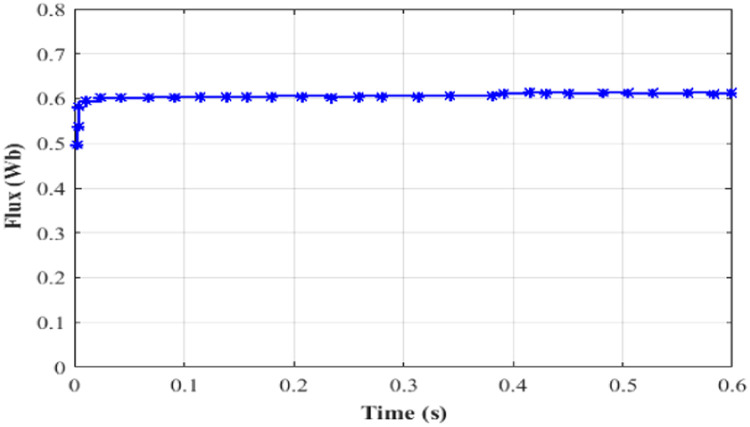
Performance of flux under test5 with PI based MRAS.

Performance of speed under test 5 with proportional integral based MRAS is depicted in Fig. 19. Here, at 0.4 s, the speed of IM is 400 rpm. Performance of torque under test5 with proportional integral based MRAS is depicted in Fig. 20. Here, the torque is 21 Nm at 0.4 s. Performance of current under test5 with PI based MRAS is shown in Fig. 21. Here, the current is occurring among −15 to 15 A during 0 to 0.4 s. Performance of flux under test5 with PI based MRAS is shown in Fig. 22.

Here, from initial to steady state, the flux is almost uniform. Comparison between PI and ANFIS based MRAS schemes is given in Table 2. In Table 3 shows the Comparison of simulation and experiment outcomes. In Table 4 shows the comparison of Torque and flux ripple. Table 5 displays the comparison of settling time.

**Table 2:** Comparison between PI and ANFIS based MRAS schemes.

Tests		Controller	
		PI	ANFIS
Test 150 rpm at no load	OS (rpm)	15	2.5
	t_s_(s)	0.28	0.05
Test 250 rpm at 50 % load torque	OS (rpm)	20	2
	t_s_(s)	100	10
Test 350 rpm to 0 to −50 rpm	OS (rpm)	15	2
	t_s_(s)	0.25	0.025
Test 4±25 rpm speed reversal	OS (rpm)	13	3
	t_s_(s)	0.22	0.025

* OS - OverShoot, US - UnderShoot and t_s_ -Settling Time.

**Table 3:** Comparison of simulation and experiment results.

Techniques	Efficiency ( %)	
	Experimental results	Simulation results
PI based MRAS	98.75	98.901
ANFIS based MRAS	99.146	99.764

**Table 4:** Comparison of Torque and flux ripple.

Switching techniques	Performance comparison			
	Reference torque	Torque ripple		Flux ripple
		Nm	Pu of mean torque	
DTC drive	1.0	0.7431	2.32	0.0199
	1.3	0.59	0.91	0.0144
	1.5	0.6156	0.8347	0.0136
	1.7	0.7855	1.13	0.0145
ANFIS	1.0	0.1962	0.2290	0.0135
	1.3	0.2084	0.1740	0.0136
	1.5	0.2499	0.27	0.0130
	1.7	0.2383	0.2072	0.0134
PI	Test 150 rpm at no load	0.2019	0.2064	0.0127
	Test 250 rpm at 50 % load torque	0.2007	0.2055	0.0133
	Test 350 rpm to 0 to −50rpm	0.1987	0.1999	0.0116
	Test 4±25 rpm speed reversal	0.1977	0.1995	0.0105
ANFIS	Test 150 rpm at no load	0.2012	0.2057	0.0117
	Test 250 rpm at 50 % load torque	0.20044	0.2045	0.0103
	Test 350 rpm to 0 to −50rpm	0.1975	0.1991	0.0101
	Test 4±25 rpm speed reversal	0.1966	0.1987	0.0109

**Table 5:** Comparison of settling time.

Parameter	PI Controller	ANFIS Controller
Speed (50 rpm)	0.61	0.49
Speed error (zero rpm)	0.69	0.49
Estimated speed (49.5 rpm)	0.61	0.49

## Conclusion

The proposed Model Reference Adaptive System (MRAS) integrated with an Adaptive Neuro-Fuzzy Inference System (ANFIS) controller demonstrates significant improvements in the performance, robustness, and stability of sensorless Induction Motor (IM) drives, especially under conditions of parameter variations such as changes in motor parameters or load torque. The primary objective of enhancing stability and reducing sensitivity to these variations has been successfully achieved through the ANFIS-based approach. The experimental and simulation results validate the superiority of the ANFIS-based MRAS over traditional methods, such as the PI-based MRAS. The ANFIS controller achieved an impressive efficiency of 99.146 % in experimental settings and 99.764 % in simulations, outperforming the PI controller, which showed efficiencies of 98.75 % and 98.901 %, respectively. This marked improvement in efficiency highlights the ANFIS controller's capability to deliver precise and reliable speed control. Moreover, the proposed system demonstrated a significant reduction in settling time across various speed scenarios, with the ANFIS controller consistently achieving a settling time of 0.49 s, notably lower than the PI controller's performance. This faster response time indicates enhanced dynamic performance and quicker adaptation to changes, leading to smoother operation and reduced error margins. Overall, the integration of ANFIS into MRAS has proven to be an effective approach for sensorless control systems, providing higher efficiency, faster response, and greater robustness against disturbances and parameter uncertainties. These advancements make the ANFIS-based MRAS a highly viable solution for applications that require precise speed control and high reliability, offering a clear advantage over traditional control methods.

## Ethics statements

In this Manuscript no, human participants or animals their data or biological material, are not involved.

## CRediT author statement

For the individual contribution of research author and co-authors as follows: “Conceptualization, Govindharaj I, Dinesh Kumar K, Balamurugan S and Karthick G; methodology, Govindharaj I; Hardware, Govindharaj I and Yazhinian S; validation, Govindharaj I, Karthick G, Dinesh Kumar K and Micheal G; formal analysis, Balamurugan S; investigation, Karthick G; resources, Govindharaj I and Rampriya R; data curation, Yazhinian S and Anandh R; writing—original draft preparation, Govindharaj I, Dinesh Kumar K and Balamurugan S; writing—review and editing, Govindharaj I and Yazhinian S; visualization, Rampriya R and Anandh R; supervision, Karthick G and Rampriya R.

## Declaration of compting interest

The authors declare that they have no known competing financial interests or personal relationships that could have appeared to influence the work reported in this paper.

## Data Availability

No data was used for the research described in the article.
